# 11β-Hydroxysteroid Dehydrogenase-1 Is a Novel Regulator of Skin Homeostasis and a Candidate Target for Promoting Tissue Repair

**DOI:** 10.1371/journal.pone.0025039

**Published:** 2011-09-20

**Authors:** Mika Terao, Hiroyuki Murota, Akihiro Kimura, Arisa Kato, Akiko Ishikawa, Ken Igawa, Eiji Miyoshi, Ichiro Katayama

**Affiliations:** 1 Department of Dermatology, Graduate School of Medicine, Osaka University, Suita, Osaka, Japan; 2 Department of Molecular Biochemistry and Clinical Investigation, Osaka University, Suita, Osaka, Japan; Cardiff University, United Kingdom

## Abstract

11β-hydroxysteroid dehydrogenase 1 (11β-HSD1) catalyzes the interconversion of cortisone and cortisol within the endoplasmic reticulum. 11β-HSD1 is expressed widely, most notably in the liver, adipose tissue, and central nervous system. It has been studied intensely over the last 10 years because its activity is reported to be increased in visceral adipose tissue of obese people. Epidermal keratinocytes and dermal fibroblasts also express 11β-HSD1. However, the function of the enzymatic activity 11β-HSD1 in skin is not known. We found that 11β-HSD1 was expressed in human and murine epidermis, and this expression increased as keratinocytes differentiate. The expression of 11β-HSD1 by normal human epidermal keratinocytes (NHEKs) was increased by starvation or calcium-induced differentiation *in vitro*. A selective inhibitor of 11β-HSD1 promoted proliferation of NHEKs and normal human dermal fibroblasts, but did not alter the differentiation of NHEKs. Topical application of selective 11β-HSD1 inhibitor to the dorsal skin of hairless mice caused proliferation of keratinocytes. Taken together, these data suggest that 11β-HSD1 is involved in tissue remodeling of the skin. This hypothesis was further supported by the observation that topical application of the selective 11β-HSD1 inhibitor enhanced cutaneous wound healing in C57BL/6 mice and *ob/ob* mice. Collectively, we conclude that 11β-HSD1 is negatively regulating the proliferation of keratinocytes and fibroblasts, and cutaneous wound healing. Hence, 11β-HSD1 might maintain skin homeostasis by regulating the proliferation of keratinocytes and dermal fibroblasts. Thus 11β-HSD1 is a novel candidate target for the design of skin disease treatments.

## Introduction

The endogenous steroid hormone glucocorticoid (GC) is released in response to various stressors such as physical injury and psychological stress. It regulates biological processes including growth, development, metabolism, and behavior [1], [2]. In mammalian cells, it induces diverse responses including differentiation, proliferation, and apoptosis [3].

GC is the most effective anti-inflammatory drug for treating acute and chronic inflammatory diseases, and has been used for more than half a century. The major anti-inflammatory mechanism of GC is the repression of inflammatory gene transcription factors such as nuclear factor κB and activator protein-1 [1], [4]. Topical application of GC ointment is one of the most common treatments for inflammatory dermatitis, and its mechanism is thought to be its anti-inflammatory effects on keratinocytes and skin infiltrating inflammatory cells. In addition to its strong anti-inflammatory effects, GC also influences keratinocyte biology in other ways. Microarray analyses have revealed that dexamethasone, a synthetic glucocorticoid, regulates genes associated with differentiation, metabolism, and inflammation in keratinocytes [5].

Cortisol is the endogenous GC in humans. The enzyme 11β-hydroxysteroid dehydrogenase (11β-HSD) is known to catalyze the interconversion between hormonally active cortisol and inactive cortisone in cells [6], [7], [8]. The two iso-enzymes of 11β-HSD both reside in the endoplasmic reticulum membrane [9]. The 11β-HSD1 isoform, which catalyzes the conversion of cortisone to cortisol, is widely expressed at the highest levels in the liver, lung, adipose tissue, ovary, and central nervous system. The 11β-HSD2 isoform, which catalyzes the conversion of cortisol to cortisone, is highly expressed in the distal nephron, colon, sweat glands, and placenta. Because 11β-HSD1 activity is reported to be elevated in the visceral adipose tissue of obese people, it has been studied intensely over the last 10 years [10], [11], [12]. Targeted overexpression of 11β-HSD1 in adipose tissue in mice has been found to model metabolic syndrome [13], [14].

Recently, 11β-HSD1 was found to be expressed in epidermal keratinocytes, dermal fibroblasts, and outer hair follicle root sheath cells. 11β-HSD1 expression increases with age in primary dermal fibroblasts and in skin tissues [15], [16]. Furthermore, Cirillo et al. demonstrated enzymatic activity of 11β-HSDs in keratinocyte in culture [17]. While these results suggested that 11β-HSDs have functions in skin component cells, the *in vivo* functions of 11β-HSDs, in skin homeostasis remained unclear.

In this study, we demonstrate that 11β-HSD1 is critical for skin homeostasis, which functions by modulating keratinocyte and fibroblast proliferation. In addition, we show the effect of topical application of a selective inhibitor of 11β-HSD1 on mouse skin and cutaneous wound healing, which collectively may demonstrate the possibility of 11β-HSD1 as a novel target in treating cutaneous disease.

## Materials and Methods

### Cell culture

Normal human epidermal keratinocytes (NHEKs) and normal human dermal fibroblasts (NHDFs) were purchased from DS Pharma Biomedical (Osaka, Japan). NHEKs were cultured on type-1 collagen-coated plates (Asahi Techno Glass, Funabashi, Japan) in human keratinocyte serum-free medium (DS Pharma Biomedical) supplemented with bovine pituitary extract. Dulbecco's modified Eagle's medium (DMEM) containing 10% fetal bovine serum (FBS) was used to culture NHDFs. Isolation and culture of mouse keratinocytes and mouse fibroblasts were carried out as previously described [18]. Full-thickness skin harvested from day 2 to day 4 newborn mice was treated with 4 mg/ml of dispase (Gibco; Invitrogen, Paisley, UK) for 1 h at 37°C. Next, the epidermis was peeled from the dermis. The epidermis was trypsinized to prepare single cells. It was then incubated in Human Keratinocyte Serum Free Medium for 6 h at 37°C under an atmosphere with 5% CO_2_. Non-adherent cells were washed away with phosphate-buffered saline (PBS) twice, and then cultured for 2–3 days in human keratinocyte serum free medium before use in experiments. The dermis was placed in PBS+0.05% type-1 collagenase (Sigma-Aldrich, St Louis, MO, USA) and incubated at 37°C for 30 min with vigorous agitation to prepare single cells. After filtration, cells were centrifuged at 200 g for 10 min, resuspended in DMEM+10% FBS, and incubated at 37°C and in 5% CO_2_. First or second passage fibroblasts were used for experiments.

### Histopathological analysis

Samples of normal skin from healthy volunteers were taken after written informed consent. All studies were approved by the ethical committee of Osaka University. Samples were fixed in 10% formaldehyde for 24 h, followed by embedding in paraffin and microtome sectioning. Slides were stained with hematoxylin and eosin (H&E). For immunohistochemical analysis, sections were hydrated by passage through xylene and graded ethanols. After antigen retrieval for 10 min at 90°C in citric buffer, pH 6.0, the slides were blocked with serum-free protein block (Dako-Cytomation, Carpinteria, CA, USA) for 10 min, then incubated with primary antibody overnight at 4°C (rabbit anti-11β-HSD1 antibody 1∶100 dilution, Abcam, Cambridge, UK; rabbit anti-Ki-67 antibody 1∶500 dilution, Novocastra Laboratories Ltd, Newcastle, UK). After washing with tris-buffered saline (TBS) containing 0.05% Triton-X100, slides were mounted using the Vectastain ABC kit® (Vector Laboratories, Burlingame, CA, USA) followed by counterstaining with haematoxylin. Rabbit IgG were used as the isotype controls. For immunofluorescent analysis, sections were hydrated as described above and incubated with primary antibody (rabbit anti-11β-HSD1 antibody 1∶100 dilution and mouse anti-keratin 14 antibody 1∶500 dilution, Abcam), followed by secondary antibody (anti-rabbit Alexa Fluor 555 and anti-mouse Alexa Fluor 488, Invitrogen).

### Western blotting

Cell samples were solubilized at 4°C in lysis buffer (0.5% sodium deoxycholate, 1% Nonidet P40, 0.1% sodium dodecyl sulphate, 100 µg/ml phenylmethylsulphonyl fluoride, 1 mM sodium orthovanadate, and protease inhibitor cocktail). For *in vivo* samples, skins were crushed in liquid nitrogen and solubilized at 4°C in lysis buffer. Ten micrograms of protein were separated on SDS-polyacrylamide gels and transferred onto polyvinylidine fluoride membranes (Bio-Rad, Hercules, CA, USA). Non-specific protein binding was blocked by incubating the membranes in 5% w/v non-fat milk powder in TBS-T (50 mM Tris-HCl, pH 7.6, 150 mM NaCl, and 0.1% v/v Tween-20). The membranes were incubated with sheep anti-11β-HSD1 antibody (The Binding Site, Birmingham, UK), rabbit anti-keratin 1 antibody (Covance, Emeryville, CA, USA), and anti-involucrin (IVL) antibody (Santa Cruz Biotechnology, Santa Cruz, CA, USA) at a dilution of 1∶1000 overnight at 4°C or with mouse monoclonal anti-β-actin (Sigma-Aldrich, St. Louis, MO, USA) at a dilution of 1∶5000 for 30 min at room temperature. Then, the membranes were washed three times in TBS-T for 5 min. Finally, the membranes were incubated with either HRP-conjugated anti-rabbit, anti-mouse, or anti-sheep antibody at a dilution of 1∶10,000 for 60 min at room temperature. Protein bands were detected using the ECL Plus kit (GE Healthcare, Buckinghamshire, UK). The intensity of the bands was quantified by using NIH image J software.

### 11β-HSD1 inhibitor treatment

11β-HSD1 inhibitor (385581) purchased from Merck (Whitehouse Station, NJ, USA) is a potent inhibitor of 11β-HSD1 with >450- and >100-fold selectivity over human and mouse 11β-HSD2, respectively [19]. The inhibitor was dissolved in DMSO and further diluted more than 100,000-fold in culture medium (for *in vitro* experiments), in a 1∶1 mixture of acetone∶olive oil (for *in vivo* topical application), or in PBS (for *in vivo* wound healing). DMSO was used as a vehicle control.

### MTS cell viability assay

Cellular viability was assessed using CellTiter96® Aqueous One Solution Cell Proliferation Assay (Promega, Madison, WI, USA). Briefly, NHEKs or NHDFs were seeded onto 96-well plates (5000 cells/well or 500 cells/well in 100 µl medium, respectively). The cells were allowed to attach for 24 h and then incubated with 11β-HSD1 inhibitor or vehicle control at the indicated doses for 48 h. Next, 20 µl of MTS reagent was added, and the cells were incubated for 2 h. Optical density was measured at 490 nm with a Micro Plate Reader (Bio-Rad, Hercules, CA, USA).

### BrdU incorporation assay

Cell proliferation was assessed using cell proliferation ELISA, BrdU (Roche, Basel, Switzerland) according to the manufacturer's protocol. Briefly, NHEKs were seeded onto 96-well plates (5000 cells/well in 100 µl medium). The cells were allowed to attach for 24 h and then incubated with 11β-HSD1 inhibitor or vehicle control at the indicated doses for 48 h. Next, cells were labeled with BrdU, and incubated for 4 h. BrdU incorporation was quantified by measuring with a Micro Plate Reader (Bio-Rad) at 450 nm.

### siRNA transfection

NHEKs (50,000 cells/ml) were seeded on type-1 collagen coated plates 1 day prior to transfection. Cells were transfected with 11β-HSD1 or control siRNAs (Invitrogen) at 50 nM using RNAi MAX (Invitrogen), and the culture medium was replaced 6 h later. Cells were used for experiments 48 h after transfection.

### RNA isolation and quantitative real time polymerase chain reaction (rtPCR)

Total RNA was isolated from cells using the SV Total RNA Isolation System (Promega). The product was reverse-transcribed into first-strand complementary DNA (cDNA). Thereafter, the expression of 11β-HSD1, 11β-HSD2, IVL, and keratin 10 (K10) was measured using the Power SYBR Green PCR Master Mix (Applied Biosystems, Foster City, CA) according to the manufacturer's protocol. Glyceraldehyde-3-phosphate dehydrogenase (GAPDH) was used to normalize the mRNA as quantified GAPDH was not affected by the treatment. Similar results were obtained in each experiment when another internal control, β-actin, was used to normalize the mRNA (data not shown). Sequence-specific primers were designed as follows: 11β-HSD1, sense: 5′-tctcctctctggctgggaaag, antisense: 5′- gaacccatccaaagcaaacttg; IVL, sense: 5′-tctgcctcagccttactgtg, antisense: 5′-ggaggaggaacagtcttgagg; K10, sense: 5′-tgaaaagcatggcaactcac, antisense: 5′-tgtcgatctgaagcaggatg; Fibroblast growth factor-2 (FGF-2), sense: 5′-agagcgaccctcacatcaag, antisense: 5′- actgcccagttcgtttcagt; TGF-β, sense: 5′- cacgtggagctgtaccagaa: 5′- gaacccgttgatgtccactt ; Matrix metalloproteinase-1 (MMP-1), sense: 5′- gtgctaaaggtgccaatggt, antisense: 5′- tccttggggtatccgtgtag ; Collagen 1 alpha 1 (Col1a1), sense: 5′- ctcctcgctttccttcctct, antisense: 5′- ctcctcgctttccttcctct ; and GAPDH, sense: 5′- ggagtcaacggatttggtcgta-3′, antisense: 5′- gcaacaatatccactttaccagagttaa-3′. Real-time PCR (40 cycles of denaturation at 92°C for 15 seconds and annealing at 60°C for 60 seconds) was run on an ABI 7000 Prism (Applied Biosystems). Samples without reverse transcriptase (negative control) did not show any amplification.

### Cortisol measurement by ELISA

NHEKs (10,000 cells/ml, 100 µl) were seeded on 96-well type-A collagen-coated plates. The cells were allowed to attach for 24 h and then the medium was changed to a high calcium (1.2 mM) basal medium that did not contain bovine pituitary extract, to remove cortisol from the culture media. The culture media were harvested 48 h later. Harvested samples were stored at −20°C until use. The amount of cortisol in samples was measured with an Cortisol EIA kit (Cayman Chemical Company, Ann Arbor, MI, USA).

### Wound healing assay

Male C57BL/6 and C57BL/6J-*ob/ob* mice were obtained from Japan Charles River, Inc. Animal care was in accordance with the institutional guidelines of Osaka University. At 6 weeks of age, dorsal hairs were removed by using hair removal cream (epilat, Kracie, Inc., Tokyo, Japan). Full-thickness 15-mm wounds were created on the backs of mice (n = 3 in each group for first experiment and n = 4 in each group for second experiment) a day after hair removal. 11β-HSD1 inhibitor (10 µM) or vehicle control dissolved in PBS was applied to the wound and the wound was covered with hydrocolloid dressing. This application was repeated every 2 days. The wound areas were calculated by measuring the major and minor axes on days, 0, 2, 4, 6, 8, 10, and 12 after wounds were created.

### Topical 11β-HSD1 inhibitor treatment

Eight-week-old male Hos: HR-1 mice (hairless mice) were obtained from Japan SLC, Inc. Animal care was in accordance with the institutional guidelines of Osaka University. Mouse dorsal skins (n = 3 in each group for first experiment and n = 5 in each group for second experiment) were treated with 11β-HSD1 inhibitor (50 µM) or vehicle control dissolved in a 1∶1 mixture of acetone∶olive oil for 5 continuous days. One day after the last treatment, the treated dorsal skins were harvested for histological analysis.

### Statistical analysis

The data are expressed as mean values ± standard deviation (SD). The unpaired Student's *t*-test was used to determine the level of significance of differences between the sample means.

## Results

### 11β-HSD1 expression in the skin

**Figure 1:**
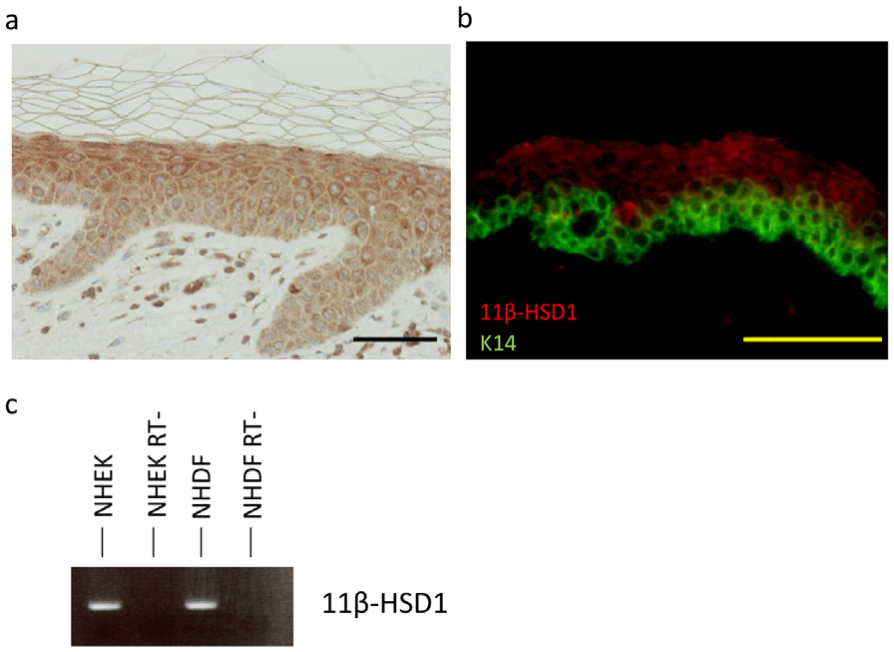
11β-HSD1 expression in human skin. (a) Immunohistochemical staining of 11β-HSD1 (DAB staining) in normal skin tissue. Bar = 50 µM (b) Immunofluorescent staining of 11β-HSD1 (red) and keratin 14 (green). Bar = 100 µM (c) PCR detecting 11β-HSD1 in NHEKs and NHDFs. RT-: samples without reverse transcriptase (negative control).

First, the expression of 11β-HSD1 in healthy skin was examined. 11β-HSD1 was broadly expressed in all layers of the epidermis and in dermal fibroblasts (Figure 1a). Its expression was stronger in the cytoplasm of supra-basal cells, and only weakly detected in basal cells. This was also confirmed by double staining with both the anti-11β-HSD1 antibody and the basal cell marker, anti-K14 (Figure 1b). The expression of 11β-HSD1 was also detected in cultured NHEKs and in NHDFs (Figure 1c).

### 11β-HSD1 expression is increased by starvation or calcium induced differentiation

**Figure 2:**
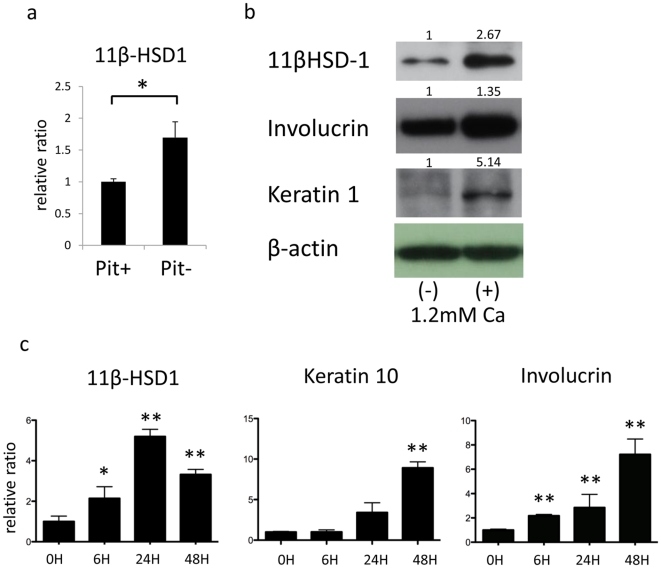
11β-HSD1 expression is increased with starvation and differentiation. (a) The relative expression of 11β-HSD1 in NHEKs assessed by rtPCR with or without pituitary extract (pit) in culture media. GAPDH was used as an internal control. (b)Western blotting for detecting 11β-HSD1, Keratin 1, and Involucrin 48 h after adding 1.2 mM of calcium to culture media of NHEKs. The numbers indicate the relative ratio to β-actin. (c) The relative expressions of 11β-HSD1, Keratin 10, and Involucrin of the indicated hour after adding 1.2 mM calcium to culture media of NHEKs assessed by rtPCR. GAPDH was used as an internal control. An asterisk indicates a statistically significant difference (*P<0.05, **P<0.01, Student's *t*-test).

We next investigated whether the starvation and differentiation alter the expression of 11β-HSD1 in NHEKs. Starving keratinocytes by depriving them of pituitary extract in the culture media retards the growth of keratinocytes. Twenty-four hours of starvation significantly increased the expression of 11β-HSD1 (Figure 2a). NHEKs are known to differentiate when 1.2 mM calcium is added. This treatment causes the early differentiation markers keratin 1 (K1), K10, and IVL to increase as the cells differentiate [20], [21]. The stimulation of differentiation with 1.2 mM of calcium increased the expression of 11β-HSD1 in NHEKs (Figure 2b and 2c). These results indicate that starvation of essential supplements or calcium-induced differentiation increases the expression of 11β-HSD1 in NHEKs.

### 11β-HSD1 regulates proliferation, but not differentiation, of NHEKs

**Figure 3:**
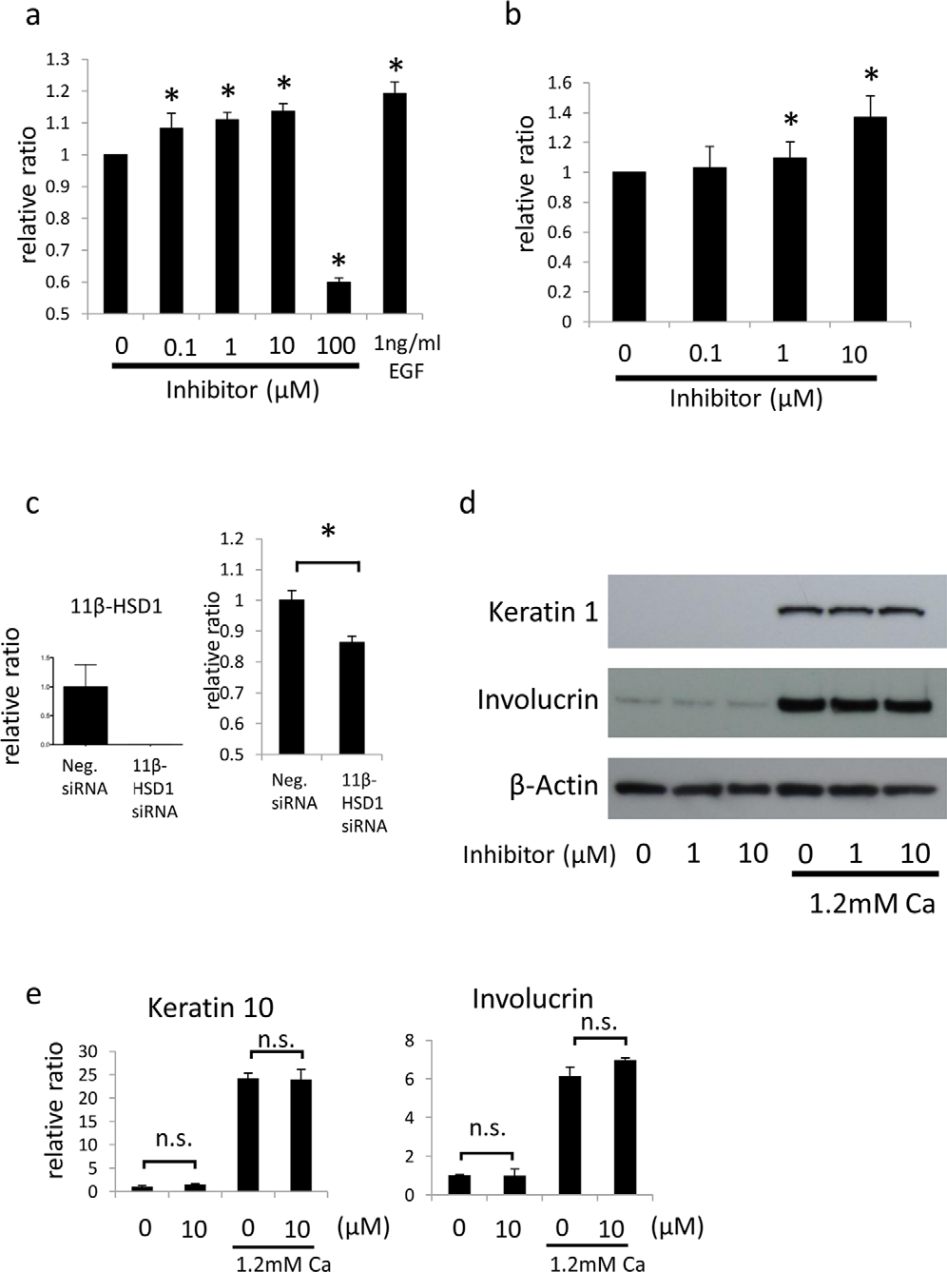
11β-HSD1 regulates proliferation but not differentiation of NHEKs. (a,b) 11β-HSD1 selective inhibitor was applied to NHEKs at indicated dose and proliferation of the cells was assessed by MTS assay (a) and BrdU absorption (b) 72 h later. DMSO was applied as vehicle control and epidermal growth factor (EGF) was used as positive control in MTS assay. The relative ratio compared with absorbance of vehicle control (0 µM) is suggested. The histograms indicate means and SDs for eight independent experiments. An asterisk (*) indicates a statistically significant difference from the vehicle treated group (P<0.05, Student's *t*-test). (c) siRNA knockdown efficacy (left) and MTS assay (right) of NHEKs transfected with 11β-HSD1 or control. Assay was performed 48 h after transfection. Transfection of si11β-HSD1 decreased the mRNA expression 11β-HSD1 more than 95% assessed by rtPCR. GAPDH was used as an internal control. The histograms indicate means and SDs for eight independent experiments. An asterisk (*) indicates a statistically significant difference from the vehicle treated group (P<0.05, Student's *t*-test). (d) Western blotting of NHEKs for detecting Keratin 1, and Involucrin treated with 11β-HSD1 selective inhibitor at indicated dose for 72 h with or without 1.2 mM calcium treatment. β-actin was used as an internal control. (e) The relative expressions of Keratin 10 and Involucrin treated with 10 µM 11β-HSD1 selective inhibitor for 48 h with or without 1.2 mM calcium treatment assessed by rtPCR. GAPDH was used as an internal control. n.s.: not significant.

To determine if 11β-HSD1 modulated keratinocyte proliferation, we investigated the effect of selective 11β-HSD1 inhibitor on the proliferation of NHEKs. Addition of 100 nM–10 µM of inhibitor to culture medium, induced cell proliferation in a dose dependent manner in both MTS assays (Figure 3a) and BrdU absorption assays (Figure 3b), suggesting that 11β-HSD1 inhibits keratinoctye proliferation. In contrast, higher doses (100 µM) of inhibitor decreased cell viability. Knocking down 11β-HSD1 with siRNA also reduced the viability of NHEKs (Figure 3c). These observations suggest that basal levels of 11β-HSD1 are essential for keratinocytes survival, and excessive loss of 11β-HSD1 activity with higher doses of inhibitor (100 µM) or siRNA-mediated depletion, can therefore not be used to evaluate the functions of 11β-HSD1 in cortisol production, proliferation, or differentiation of keratinocytes.

Next, we evaluated the effects of 11β-HSD1 inhibitor on the calcium-stimulated differentiation of NHEKs. Although calcium treatment increased the expression of 11β-HSD1, protein and mRNA for K1 or K10, and IVL were not affected by 1 to 10 µM of selective 11β-HSD1 inhibitor (Figure 3d,e). These results indicated that 11β-HSD1 might be involved in the proliferation but not in the differentiation of NHEKs.

### 11β-HSD1 regulates proliferation of NHDFs

**Figure 4:**
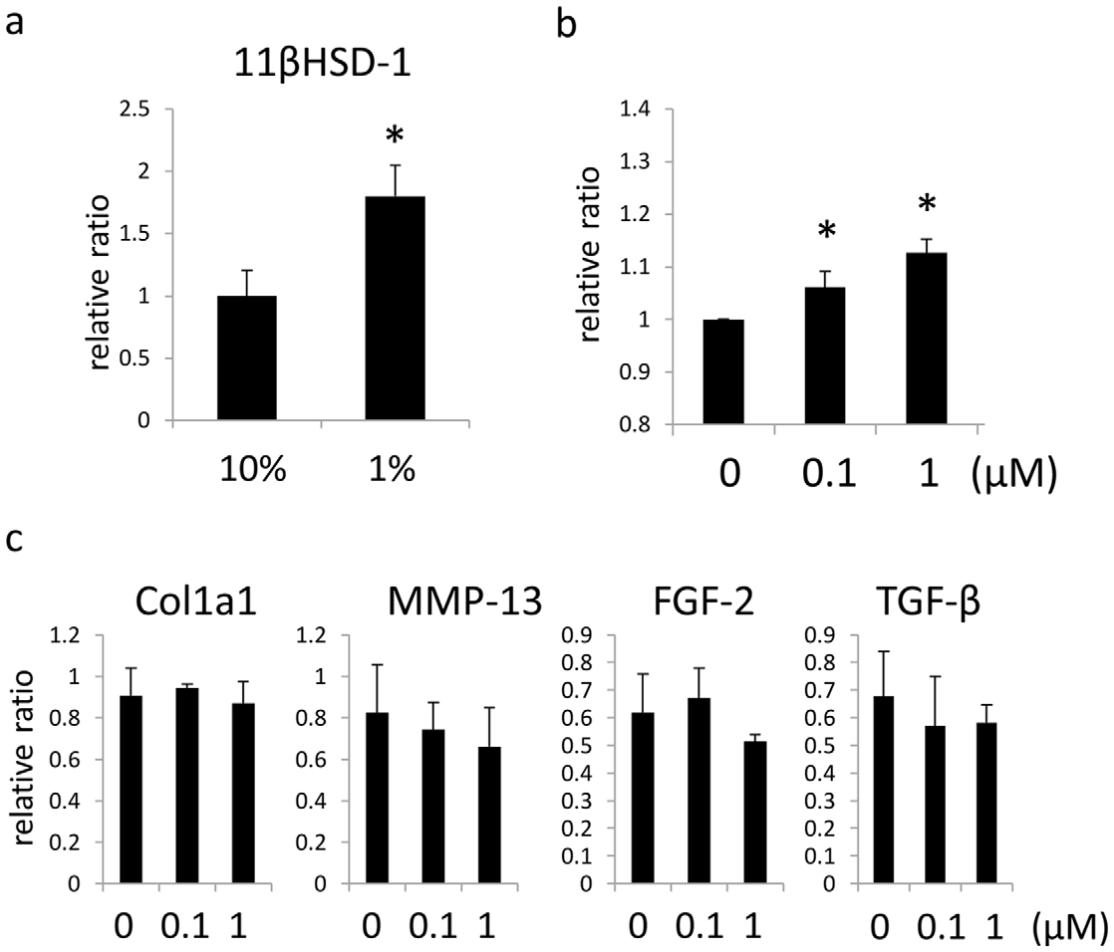
11β-HSD1 regulates proliferation of NHDFs. (a) The relative expression of 11β-HSD1 in NDHF assessed by rtPCR with 10% FBS or 1% FBS in culture media. GAPDH was used as an internal control. (b) 11β-HSD1 selective inhibitor was applied to NHDFs cultured in DMEM containing 2% FBS at indicated dose and proliferation of the cells was assessed by MTS assay 72 h later. DMSO was applied as vehicle control. The histograms indicate means and SDs for eight independent experiments. An asterisk (*) indicates a statistically significant difference from the vehicle treated group (P<0.05, Student's *t*-test). (c) The relative expressions of Col1a1, MMP-1, FGF-2, TGF-β treated with 11β-HSD1 selective inhibitor at indicated dose for 48 h assessed by rtPCR. GAPDH was used as an internal control.

We next investigated the function of 11β-HSD1 in NHDFs. Starving NHDFs by reducing medium concentrations of FBS from 10% to 1% for 24 h retards cell growth. The expression of 11β-HSD1 was significantly enhanced in starvation conditions (Figure 4a). Furthermore, similarly to the effects on keratinocytes, the selective 11β-HSD1 inhibitor at doses of 100 nM and 1 µM induced proliferation of NHDFs, demonstrating that 11β-HSD1 also negatively regulates NHDFs proliferation (Figure 4b). Next, the effect of 11β-HSD1 inhibitor on the expression of fibrogenic cytokines and fibroblast growth factors was evaluated (Figure 4c). However, inhibition of 11β-HSD1 at these doses did not affect the expression of Col1a1, MMP-13, TGF-β, or FGF-2. This indicates that 11β-HSD1 was not involved in collagen metabolism, and inhibits the proliferation of NHDFs via pathways independent of the autocrine effects of these cytokines and growth factors.

### Topical application of 11β-HSD1 inhibitor induces hyperproliferation of the epidermis

**Figure 5:**
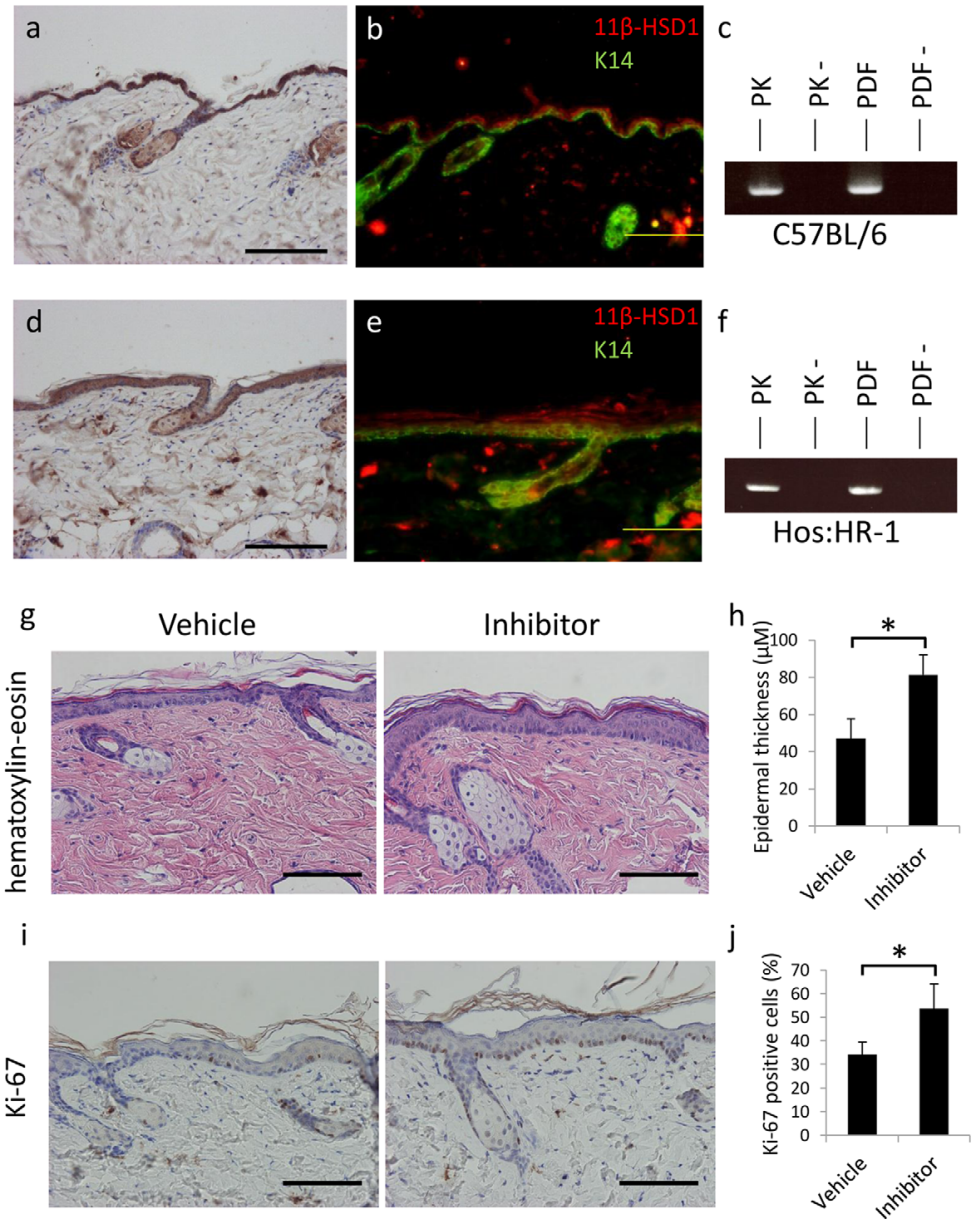
Selective inhibitor of 11β-HSD1 proliferates keratinocytes in murine skin. (a, d) Immunohistochemical staining of 11β-HSD1 (DAB staining) in C57BL/6 mouse (a) and Hos: HR-1 (hairless) mouse (d) skin tissue. Bar = 50 µM. (b, e) Immunofluorescent staining of 11β-HSD1 (red) and keratin 14 (green) in C57BL/6 mouse (b) and Hos: HR-1 mouse (e) skin tissue. Bar = 100 µM. (c, f) PCR detecting 11β-HSD1 in primary mouse keratinocytes and primary mouse dermal fibroblasts of C57BL/6 mouse (c) and Hos: HR-1 mouse (f). RT-: samples without reverse transcriptase (negative control). (g–j) Representative H&E staining (g) and Ki-67 staining (i) of 11β-HSD1 selective inhibitor or vehicle (1∶1, acetone∶olive oil) treated skin of Hos: HR-1 mice. Bar = 100 µm. (h) Epidermal thickness of vehicle and inhibitor treated mice. Intrafollicular epidermal thickness was calculated by averaging five locations in each section. Three sections from each mouse were evaluated. Bars show mean epidermal thickness ± SD of vehicle-treated mice (n = 5) and inhibitor-treated mice (n = 5; *P<0.01, Student's *t*-test). (j) The percentage of Ki-67 positive cells. Analyses were performed by counting the total number of basal cells and cells expressing nuclear Ki-67 stain. Three sections from each mouse were evaluated. Bars indicate mean ± SD of vehicle-treated mice (n = 5) and inhibitor-treated mice (n = 5; *P<0.05, Student's *t*-test).

To investigate the function of 11β-HSD1 *in vivo*, hairless mouse skin was exposed to 11β-HSD1 inhibitor. 11β-HSD1 is also expressed in the epidermis and fibroblasts of murine skin in C57BL/6 mice and Hos: HR-1 (hairless) mice (Figure 5a,b,d,e). The expression of 11β-HSD1 was also detected in cultured primary mouse keratinocytes and in cultured primary dermal fibroblasts derived from C57BL/6 and Hos: HR-1 mice (Figure 5c,f). Application of 50 µM selective 11β-HSD1 inhibitor to the dorsal skin of Hos: HR-1 mice for five continuous days induced acanthosis (Figure 5g). The epidermal thickness was significantly higher in selective 11β-HSD1 inhibitor treated groups than control groups (Figure 5h). In addition, the number of Ki-67 positive cells was significantly higher in 11β-HSD1 inhibitor treated skin than in vehicle treated skin (Figure 5i,j). These results demonstrate that 11β-HSD1 inhibitor also promotes the proliferation of keratinocytes *in vivo*.

### 11β-HSD1 inhibitor promotes wound healing in C57BL/6 mice

**Figure 6:**
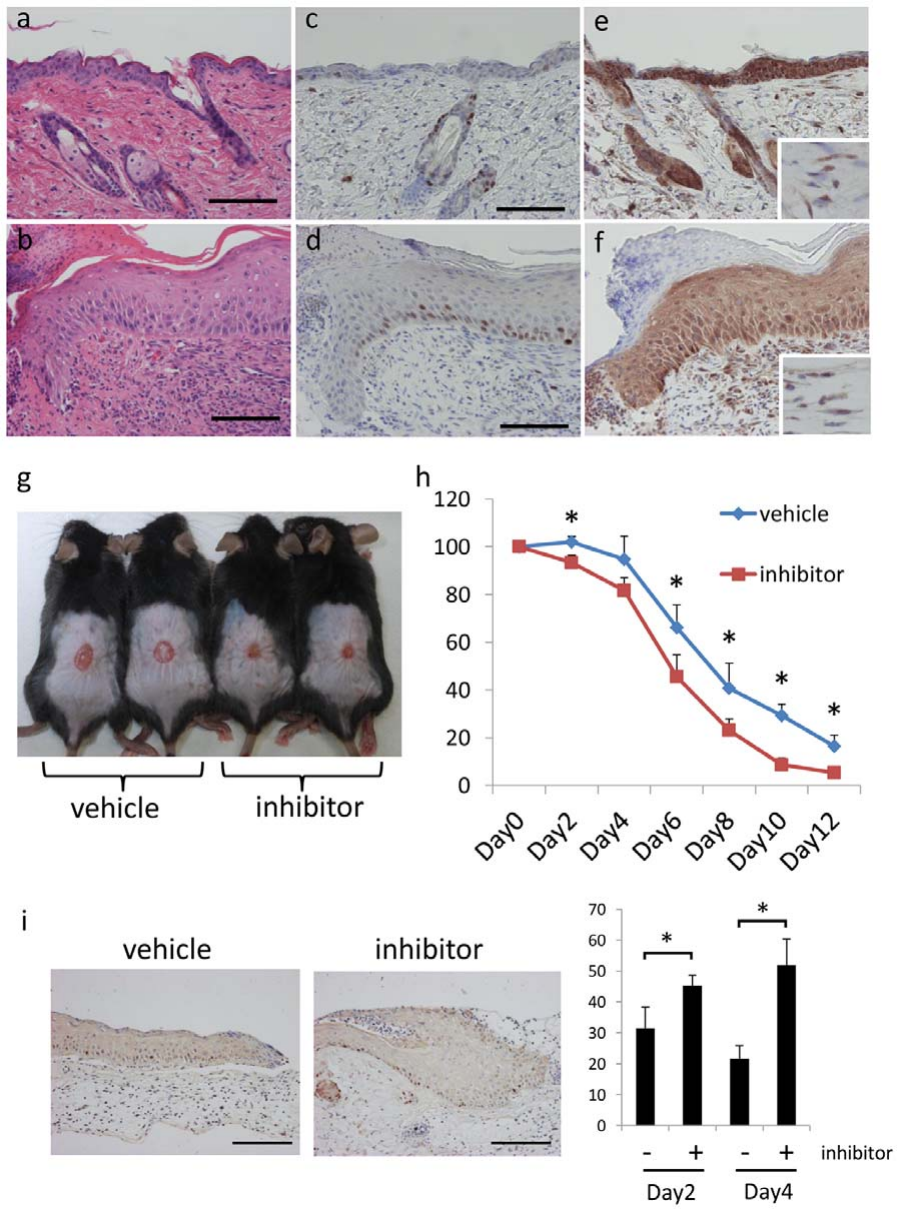
The role of 11β-HSD1 in wound healing of C57BL/6 mice. (a–f) H&E (a, b), Ki-67 (c, d), and 11β-HSD1 (e, f) staining of ulcer edge and non ulcer skin of the same section. Inserts: high magnification of the fibroblasts. Bar = 100 µm. (g) Macroscopic view of wound healing on day 10. A 15-mm wound was created on the back of 6-week-old male mice and wound closure was monitored with application of vehicle or 11β-HSD1 inhibitor every other day. (h) Reduction of wound area on days 2, 4, 6, 8, 10, and 12. The histograms indicate means and standard deviations for four mice in each group. An asterisk indicates a statistically significant difference (*P<0.05, Student's *t*-test). (i) Representative Ki-67 staining in day2 wound edge skin and the percentage of Ki-67 positive cells in day2 and day4 wound edge epidermis. Analyses were performed by counting the total number of basal cells and cells expressing nuclear Ki-67 stain. Bars indicate mean ± SD of vehicle-treated mice (n = 6) and inhibitor-treated mice (n = 6; *P<0.05, Student's *t*-test). Bar = 100 µm.

Taken together, these findings demonstrate that 11β-HSD1 regulates the proliferation of keratinocytes and fibroblasts. We therefore hypothesized that 11β-HSD1 inhibitor would promote wound healing. The keratinocytes at wound edges are hyperproliferative, thus the epidermis becomes thick in this region, with increased Ki-67 positive cells (Figure 6a–d). Interestingly, the intensity of 11β-HSD1 detected with immunohistochemical staining was lower in wound edge keratinocytes than in non wound keratinocytes in the same section (Figure 6e,f). The intensity of 11β-HSD1 did not differ between wound edge fibroblasts and non-wound fibroblasts (Figure 6e,f inserts). Because our data show that 11β-HSD1 negatively regulates the proliferation of keratinocytes, we considered that the decreased expression of 11β-HSD1 in wound edge keratinocytes might be promoting their proliferative state. To investigate whether selective 11β-HSD1 inhibitor could promote wound healing, we applied 10 µM 11β-HSD1 inhibitor every other day to wounds created on the dorsal skin of C57BL/6 mice. The wound areas were significantly smaller in the 11β-HSD1 inhibitor treated group than the vehicle treated group (Figure 6g,h). The number of Ki-67 positive cells was significantly higher on day2 and day4 wound edge epidermis in the 11β-HSD1 inhibitor treated group than the vehicle treated group (Figure 6i).

### 11β-HSD1 inhibitor promotes wound healing in *ob/ob* mice

**Figure 7:**
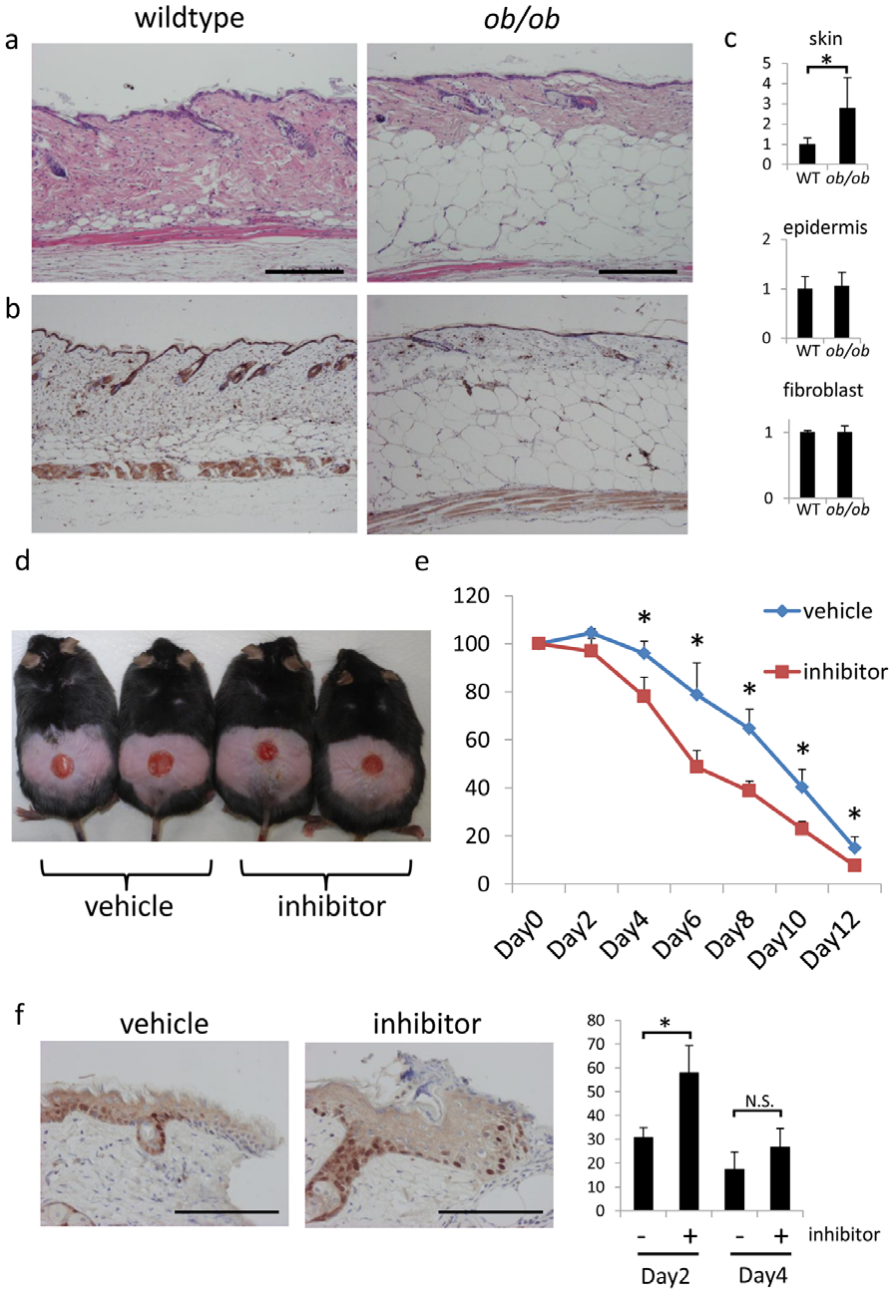
Selective 11β-HSD1 inhibitor enhance wound healing in *ob/ob* mice. (a,b) Representative H&E staining (a) and 11β-HSD1 staining (b) of 6-week-old male wildtype and *ob/ob* mice. Bar = 50 µm. (c) The relative expressions of 11β-HSD1 in epidermis, fibroblasts, and whole skin extract of wildtype and *ob/ob* mice assessed by rtPCR. GAPDH was used as an internal control (P<0.05, Student's *t*-test). (d) Macroscopic view of wound healing on day 8. A 15-mm wound was created on the back of 6-week-old male *ob/ob* mice and wound closure was monitored with application of vehicle or 11β-HSD1 inhibitor every other day. (e) Reduction of wound area on days 2, 4, 6, 8, 10, and 12. The histograms indicate means and standard deviations for four mice in each group. An asterisk indicates a statistically significant difference (*P<0.05, Student's *t*-test). (f) Representative Ki-67 staining in day4 wound edge skin and the percentage of Ki-67 positive cells in day2 and day4 wound edge epidermis. Analyses were performed by counting the total number of basal cells and cells expressing nuclear Ki-67 stain. Bars indicate mean ± SD of vehicle-treated mice (n = 3) and inhibitor-treated mice (n = 3; *P<0.05, Student's *t*-test). Bar = 100 µm.

We finally assessed wound healing in obese/obese (*ob/ob*) mice, the model of impaired wound healing. In *ob/ob* mice, the dermal layer was thinner, and the subcutaneous adipose layer was thicker, than in age-matched wildtype mice (Figure 7a). Interestingly, the expression of 11β-HSD1 was significantly higher in the skin extract of *ob/ob* mice, however, the expression did not differ in the epidermal extract and the fibroblast extract (Figure 7b,c). These data suggest that increased subcutaneous adipose tissue in *ob/ob* mice is responsible for increased expression of 11β-HSD1 in the skin extract. Notably, application of 10 µM 11β-HSD1 inhibitor every other day improved wound healing more in *ob/ob* mice than in C57BL/6 mice (Figure 7d and 7e). The number of Ki-67 positive cells was significantly higher on day2 wound edge epidermis in the 11β-HSD1 inhibitor treated group than the vehicle treated group (Figure 7f).

## Discussion

The present study shows that 11β-HSD1 is a regulator of keratinocyte and fibroblast proliferation. We found that the expression of 11β-HSD1 is higher in the cytoplasm of supra-basal differentiating cells than in basal proliferating cells of the normal epidermis, and that the inhibition of 11β-HSD1 increases the proliferation of keratinocytes and fibroblasts. We also report that topical application of a selective 11β-HSD1 inhibitor promotes keratinocyte proliferation and wound healing.

Skin is one of the most chronically stress-loaded tissues because it faces the outside environment and is exposed to stressors including bacteria, ultraviolet radiation, and mechanical stimulation. Thus, it makes intuitive sense that skin expresses the functional cortisol activating enzyme 11β-HSD1. Specifically, our experiments using immunofluorescence staining revealed that 11β-HSD1 is expressed in the supra-basal area of the epidermis. This expression pattern of 11β-HSD1 is different from previous reports [17]. However, 11β-HSD1 expression being limited to the supra-basal epidermal area seems reasonable, considering that 11β-HSD1-mediated suppression of excessive proliferation in differentiated keratinocytes might contribute to maintain adequate epidermal thickness. In addition to its known anti-inflammatory properties, glucocorticoid (e.g., cortisol and corticosterone) is known to regulate the proliferation of keratinocytes and prolong epidermal turnover time [22], [23], [24], [25]. Consistent with this, we have shown that selective inhibition of 11β-HSD1 promotes the proliferation of keratinocytes both *in vitro* and *in vivo*, suggesting that intracellular activators of cortisol would negatively regulate keratinocyte proliferation (Figure 3 and 5). Hence, we conclude that topical application of selective 11β-HSD1 inhibitor has the potential to be an effective treatment to stimulate the proliferation of keratinocytes. However, we observed that high doses of selective 11β-HSD1 inhibitor and siRNA knock down of 11β-HSD1 decreased the viability of keratinocytes. Thus, it is important to determine the optimal dosage to stimulate proliferation without unwanted toxic effects. Unexpectedly, the selective 11β-HSD1 inhibitor did not influence calcium-induced differentiation of keratinocytes. As calcium-induced differentiation *in vitro* differs from *in vivo* differentiation, further study may needed to determine if 11β-HSD1 plays a functional role in keratinocyte differentiation.

Glucocorticoids are known to increase in response to stress or medical therapy, and impair wound healing because they inhibit proliferation of cells and proinflammatory cytokine production [26], [27]. In this study, we showed that 11β-HSD1 inhibitor significantly promotes cutaneous wound healing. We think the decrease in the expression of 11β-HSD1 in keratinocytes at wound edges might be a normal physiological mechanism that promotes the proliferation of keratinocytes during wound healing. Thus, the selective 11β-HSD1 inhibitor might promote wound healing because it supports this mechanism. The selective 11β-HSD1 inhibitor also promotes the proliferation of NHDFs *in vitro*, and the effect of the inhibitor on fibroblasts also might assist wound healing. The effect of inhibitor on endothelial cells and inflammatory cytokines, which also are important factors in wound healing, needs to be evaluated in the future.

It is intriguing that the inhibitor has a stronger effect on wound healing in *ob/ob* mice, a model of impaired wound healing. These mice exhibit severe diabetes and obesity syndromes, phenotypes mediated by the loss of the ob gene product: the 16 kDa cytokine leptin [28], [29]. The expression of 11β-HSD1 is elevated in stromal vascular cells and mature adipocytes isolated from the adipose tissue of *ob/ob* mice [30]. Interestingly, the expression of 11β-HSD1 was also elevated in the skin extract of *ob/ob* mice (Figure 7c). The selective 11β-HSD1 inhibitor promoted wound healing in *ob/ob* mice, almost to the same level as the inhibitor treated group of C57BL/6 mice. Thus, we hypothesize that increased expression of 11β-HSD1 in *ob/ob* mouse skin might play an important role in delayed wound healing in *ob/ob* mice. The mouse skin extract is composed of epidermis, dermis, subcutaneous adipose tissue, and cutaneous muscular tissue. It was recently reported that subcutaneous adipose tissue is an important regulator of dermal fibroblast proliferation in high-fat diet induced obese mice [31]. It is possible that not only keratinocytes and fibroblasts, but also the subcutaneous adipose layer, which is markedly increased in *ob/ob* mice, could be a source of 11β-HSD1 in *ob/ob* mice as the expression of 11β-HSD1 did not differ in the epidermal extract and the fibroblast extract. We think that the 11β-HSD1 inhibitor might also act on the subcutaneous adipose tissue to accelerate wound healing in *ob/ob* mice, although further study is needed to test this theory.

Obesity is a global problem that affects 400 million adults worldwide [12], [32]. Adipose tissue overexpression of 11β-HSD1 is observed in human obesity, and inhibition of 11β-HSD1 has been proposed to be of potential therapeutic benefit to patients with obesity and type 2 diabetes mellitus [33], [34], [35]. Our results suggest that in addition to systemic administration of 11β-HSD1 inhibitor, topical application of 11β-HSD1 inhibitor is potentially effective for the treatment of the chronic wounds of obese and diabetic patients.

In summary, the present study identifies a novel role for 11β-HSD1 in the promotions of keratinocyte and fibroblast proliferation. Targeting 11β-HSD1 could be a novel approach to treat chronic wounds, and skin diseases with aberrant proliferation.
